# Building a Runway to Subjective Happiness: The Role of Fashion Modeling Classes in Promoting Seniors’ Mental Well-Being

**DOI:** 10.3390/ijerph182413144

**Published:** 2021-12-13

**Authors:** Joon-Ho Seon, Sun-Ok Jung, Kyu-Hye Lee

**Affiliations:** 1 Human-Tech Convergence Program, Department of Clothing and Textiles, Hanyang University, Seoul 04763, Korea; jhseon@hanyang.ac.kr; 2 Department of Clothing and Textiles, Hanyang University, Seoul 04763, Korea; smodeljde@naver.com

**Keywords:** fashion model classes, flow experience, confidence in fashion, appearance satisfaction, subjective happiness

## Abstract

This study focuses on senior citizens who are participating in fashion modeling classes. Modeling classes are one of the most prominent educational leisure activities that has recently garnered the attention of the elderly population. The effect of flow experience in modeling classes on perceived happiness in life was considered. Since the activities related to modeling classes are related to fashion and appearance, the mediating roles of confidence in fashion and appearance satisfaction were included as hypotheses. Survey data of 168 women aged 50 years or older were analyzed using the PROCESS macro for SPSS and SmartPLS. Flow experience was found to have a positive effect on subjective happiness, confidence in fashion, and appearance satisfaction. However, confidence in fashion had a significant impact on appearance satisfaction but not on subjective happiness. Appearance satisfaction also had a significant effect on subjective happiness. Appearance satisfaction had a significant mediation effect from flow experience to subjective happiness, but confidence in fashion did not have significant influence in the process. However, the serial multiple mediation effect through confidence in fashion and appearance satisfaction was detected to be significant from flow experience to subjective happiness. The results are expected to help establish the direction of leisure education programs for seniors and bring progress to the research agenda on the impact of fashion confidence and appearance satisfaction on senior citizens.

## 1. Introduction

An aging population is accompanied by various problems such as the deterioration of physical and mental health, as well as that of their social environment. In addition to problems resulting from physical aging, seniors also experience psychological problems such as depression, sleep disorders, and lethargy. As it is urgent to prepare solutions for various social and psychological problems caused by an aging population, attention has been paid to the leisure activities of seniors. According to a study by Im et al. [1], participation in activities after retirement has a positive association with health and social development. Leisure activities such as dancing and physical education improve self-efficacy and self-esteem, thus improving people’s quality of life and satisfaction [2]. As the relationship between life and leisure activities after retirement is being re-examined, there is a greater need for research related to the field [3].

Recently, fashion model training classes have been highlighted as unique activities for seniors. Middle-aged and elderly seniors who started modeling as part of their daily activities gradually became professionals, and in the process, it was reported that they rediscovered their egos or experienced improved levels of confidence and pride [4]. Therefore, in this study, the fashion model training course was explored as a leisure activity that fosters personal self-esteem and creates a renewed sense of purpose due to various factors such as communication with instructors and fellow trainees, the educational environment, and its benefits. According to a previous study, individuals who experienced professional training showed higher self-esteem, confidence in fashion coordination, and physical satisfaction. Therefore, experiencing professional education may have an influence on the individual [5]. Since activities for seniors have therapeutic effects, participating in fashion modeling classes can increase confidence in fashion and satisfaction with appearance for senior citizens.

Therapy refers to the pursuit of psychological change by resolving mental illness through treatment without drugs or surgery [6]. Fashion therapy is a psychological treatment method that raises personal self-esteem and reduces negative emotions by improving people’s appearance through clothing [7]. Recently, various therapy programs have been developed, such as a beauty therapy course combining herbal medicine and beauty, deep sleep therapy, and emotional healing therapy through communication with AI [8]. However, therapy research has been conducted primarily in terms of fashion therapy on patients and retail therapy through shopping. 

This study focuses on the therapeutic aspects of the senior modeling classes. The flow experience, in particular, was assumed to be the antecedent to the therapeutic effect. According to Park and Kim [9], the flow experience onstage affects stage satisfaction, which satisfaction increases as the flow deepens. In addition, the experience of flow in physical activities has been shown to affect the perceived quality of life improvement [2]. The aim of this study was to examine the role of the flow experience during modeling classes in improving perceived happiness in life. Since the class is about fashion, the mediating role of confidence in fashion and satisfaction with appearance was investigated. 

## 2. Theoretical Background

### 2.1. Flow Experience in Modeling Classes

Flow is a concept that represents a psychological state in which an individual has an optimal experience by being completely immersed in his or her actions. When this experience interacts with people’s individual experiences, it is called a flow experience [10,11]. In other words, flow refers to a psychological state in which one is absorbed in something to the extent that one’s concentration is not shaken by any other external factors. However, in empirical studies many terms other than “flow” such as “commitment” have been confusingly used to describe the immersion [12]. Commitment refers to a state in which the will and attitude to continue to participate in the related behavior by forming an attachment to an object are created [13]. Semantically, the two terms are similar in that they indicate a state of concentrating on an action, but there is a subtle conceptual difference. For example, the construct of “flow” refers to a momentary psychological state of unintentional and natural immersion in the pleasure experienced through activities. Conversely, “commitment” expresses an interest in continuing to participate in or desiring the activity [12]. Based on the results of previous studies that show that people’s psychological self-esteem was improved in the process of participating in leisure activities [1], this study was conducted using Csikszentmihalyi’s flow theory.

To experience flow, the premise is that an individual must participate in an activity voluntarily and must enjoy the activity [10]. In other words, voluntary participation for a clear purpose can be said to accompany the pleasure of achieving the goal, and in the process, flow is experienced. In addition, if an individual’s ability to perform the activity is greater than the difficulty level of the activity, he/she will feel bored, whereas if the goal is too hard to achieve compared to the participant’s ability, he/she may experience anxiety. Flow experience frequently occurs in activities considered as leisure, and satisfactory flow experience in a leisure environment also acts as a motive for continuous participation [14]. In particular, challenging physical activities such as dance, yoga, golf, and aerobics are highly likely to be accompanied by the pleasure of achieving goals, providing an optimal environment for experiencing flow.

Flow is a factor that allows us to experience pleasant emotions and ultimately affects the subjective sense of well-being [15]. According to a study by Ragheb and Griffith [16] in which elderly people (55 years and above) were asked to rate the degrees and levels of participation in various leisure activities, for elderly people, the more they participate in in leisure activities, the more they are satisfied with leisure activities leading to a positive effect on quality of life. It was also found that flow experience in leisure and leisure satisfaction are closely related to life satisfaction [17]. In addition, there is a positive relationship between the degree of involvement in serious leisure activities, life satisfaction, and physical and mental health, and commitment has been reported to contribute to this [18]. Kim [2] found that flow in leisure activities fully mediated between participating in leisure activities and perceived happiness. Attitudes toward leisure activities were significantly mediated by flow experience in improving subjective happiness [19]. In a study by Standridge et al. [20], flow experience was heightened as participants in daily leisure activities achieved challenging goals and focused on the task at hand. They took qualitative method and conducted purposeful in-depth interviews with 12 retirees who resided in an affluent care community to measure flow experience in leisure activities. In contrast, in the case of Lee and Kim [21], flow did not have a significant effect on happiness in life. Lee and Kim collected data from college students and used a revised scale based on Csikszentmihalyi [10] to measure “serious” leisure activities. By comparing these two studies, the flow experience might not always have a positive influence on one’s happiness in life. However, the motivation of leisure activities might have affected the study results as well as the differences in age groups in each study. 

A challenging flow experience in activities satisfies a variety of needs that cannot be met in other areas of life [22]. In particular, this flow is a factor that causes positive emotions to be experienced and affects an individual’s subjective sense of well-being [15]. In most previous studies, the relationship between the experience of flow in leisure activities and the quality of life or subjective happiness has been investigated [16,17,20]. Therefore, the following hypothesis was set.

**Hypothesis** **1.** *The flow experience of seniors in modeling classes has a direct positive effect on subjective happiness*.

### 2.2. Confidence in Fashion

By participating in leisure activities, seniors can reaffirm their purpose in life, express their individual abilities, or identify important elements of life [23]. Previous studies have discussed self-confidence as an antecedent to the sense of achievement and satisfaction [24]. Confidence refers to a state of mind with which an individual can successfully accomplish a certain task and is a self-concept of one’s own importance and value [9]. Such confidence can be said to be the main knowledge required for the smooth job performance of a fashion model. For fashion models, an expectation of a successful stage performance can maximize their expressive abilities during modeling [9]. One type of educational training provided by senior modeling classes is stage walking. Such experience can improve self-efficacy and self-esteem of senior participants [5]. 

Fashion models should be confident in their fashion senses in styling and coordination of items. Confidence in fashion refers to the creation of unity and balance by appropriately arranging elements such as clothing images, materials, patterns, and accessories in consideration of color and body shape [25]. Park and Choo [26] empirically analyzed the effects of consumer confidence and confidence in fashion coordination as important factors influencing consumers’ positive attitudes toward used fashion products and purchase intentions. Studies related to the senior fashion modeling classes reported an increase in participants’ confidence in fashion coordination [5].Therefore, seniors’ confidence in fashion that can be experienced through education is considered to be a valid antecedent affecting subjective happiness. Some of the prior research reported the association of flow experience with confidence [27]. Kim and Han [28] examined the relationship between self-efficacy and dance flow experience. It was found that as the flow experience deepened, dancers became more confident in their own actions. Therefore, the following hypothesis was set.

**Hypothesis** **2-1.** *The flow experience of seniors in modeling classes has a positive effect on confidence in fashion*.

Confidence in performing any task is closely related to one’s perceived happiness [29]. Choi et al. [24] investigated the effect of confidence in dance on dance happiness. It was reported that social support among confidence factors had a significant effect on all dance happiness factors (achievement, performance saturation, self-realization, and sense of commission). Additionally, confidence in one’s ability significantly influenced one’s self-realization. By increasing ability to perform physical activity, such as stage walking, confidence in fashion may lead to a sense of happiness [24]. Therefore, the following hypothesis was set.

**Hypothesis** **2-2.** *Confidence in fashion has a positive effect on subjective happiness*.

According to a study of college students participating in Taekwondo, passion and confidence not only had a positive effect on happiness, but it was also revealed that confidence partially mediates passion and happiness [30]. Considering that the passion variable is a variable characterized by focusing on leisure activities based on an active attitude, it is similar to the flow experience variable. Therefore, it was considered that confidence in the fashion of trainees who actively participate in their modeling classes could mediate the relationship between flow experience and subjective happiness. The following hypothesis was set.

**Hypothesis** **2-3.** *Confidence in fashion significantly mediates the influence of senior’s flow experience in modeling classes on subjective happiness*.

### 2.3. Seniors’ Satisfaction with Their Appearance

Appearance refers to an individual’s external look, including clothes, body care, makeup, and hairstyle [31], and it can be said to be the fastest and most clear means of characterizing an object. It has been mentioned as an important interpersonal relationship factor in that it is the first inter-cognitive cue used when forming impressions [32]. Appearance satisfaction, which indicates the degree of self-satisfaction with one’s appearance, is a concept related to body satisfaction and tends to be recognized by an individual’s subjective evaluation [33]. The way an individual evaluates his/her appearance is influenced by the sociocultural standards of the time [34]. 

Appearance satisfaction is closely related to social activities, and the negative effects of aging anxiety and appearance dissatisfaction on quality of life can be prevented through them. In general, most activities take place through social interactions with others, so participants in leisure activities become conscious of their appearance as seen by others [35]. In this regard, the higher the level of flow experience in any activity, the higher their satisfaction with their appearance is. 

Some previous studies have focused on flow experience as a parameter to explain the mediating role between variables, while other studies have highlighted the direct effect on satisfaction [2,19]. This suggests that flow experience can play an independent role forming satisfaction. Senior modeling classes actively utilize body expressions, and participants showed higher body satisfaction and physical efficacy than non-experienced subjects [5]. Considering that appearance satisfaction is a concept deeply related to the body satisfaction, it is expected that appearance satisfaction is influenced by the level of flow experienced by the participants. The following hypothesis was set.

**Hypothesis** **3-1.** *The flow experience of seniors in modeling classes has a positive direct effect on appearance satisfaction*.

Mathes and Kahn [36] suggested that higher attractiveness is correlated with lower neuroticism and higher self-esteem of female college students. Low self-esteem can cause psychological problems such as depression, stress, and lack of social skills [37]. Park and Kim [9] examined the effects of fashion model self-expression, stage confidence, and flow satisfaction and found that both psychological and physical confidence had a positive effect on satisfaction. Confidence of an individual has a positive effect on satisfaction. The following hypothesis was established.

**Hypothesis** **3-2.** *Confidence in fashion has a positive direct on appearance satisfaction*.

Psychological problems caused by dissatisfaction with one’s appearance, often caused by physical aging, are frequently reported in studies related to seniors. The driver that causes dissatisfaction with appearance for seniors is closely related to physical aging. The fear of experiencing symptoms related to the aging process is called aging anxiety, and it is seen as a factor that negatively affects people’s psychological well-being and quality of life [38,39]. Kim and Kim [40] stated that the appearance-oriented atmosphere in modern society, which values youth, creates anxiety about aging and acts as a negative factor in individual emotions. In fact, elders have a higher fear of loss and psychological instability than other age groups, and the lower the fear of loss, the higher their life satisfaction [40]. A study by Hamermesh and Abrevaya [41] confirmed that appearance increases the feeling of happiness. Other studies [37,42] reported the significant influence of appearance satisfaction on perceived quality of life and happiness. When humans perceive their self-image positively, not only can their satisfaction with social activities increase, but they can also experience a greater sense of happiness [31]. Jeon [35] reported that appearance satisfaction and leisure activity quality had a significant effect on overall quality of life. Therefore, the appearance satisfaction of participants of physical activities such as modeling classes influences subjective happiness in life. The following hypothesis was set.

**Hypothesis** **3-3.** *Appearance satisfaction has a positive direct effect on subjective happiness*.

The theoretical review indicates that satisfaction has a direct effect on happiness, but it can be inferred from some previous studies that it can also play a mediating role. In a study by Park and Chung [43], it was found that self-assertion had a significant effect on appearance satisfaction and showed only a partially significant effect on psychological well-being. However, appearance satisfaction had a significant effect on overall psychological well-being. This means that assertiveness and appearance satisfaction have a causal relationship, suggesting that appearance satisfaction can be used as a parameter linking the relationship between assertiveness and psychological well-being. Therefore, it was expected in this study that appearance satisfaction would act as a sufficient parameter that influences the causation from flow experience and subjective happiness. Additionally, research has shown that consumer self-confidence in consumption plays a mediating role linking independent variables and consumer life satisfaction [44], indicating that confidence in fashion is expected to be a significant mediator in the relationship between flow experience in modeling classes and appearance satisfaction. Therefore, it was determined in this study that there could be a multiple mediation effect in which confidence in fashion and appearance satisfaction sequentially affect subjective happiness. The following hypotheses were established for empirical verification.

**Hypothesis** **3-4.** *Appearance satisfaction mediates the effects from flow experience and subjective happiness*.

**Hypothesis** **3-5.** *Confidence in fashion and appearance satisfaction sequentially mediate the effects from flow experience and subjective happiness*.

## 3. Research Methods and Procedures

### 3.1. Conceptual Model Depiction

**Figure 1:**
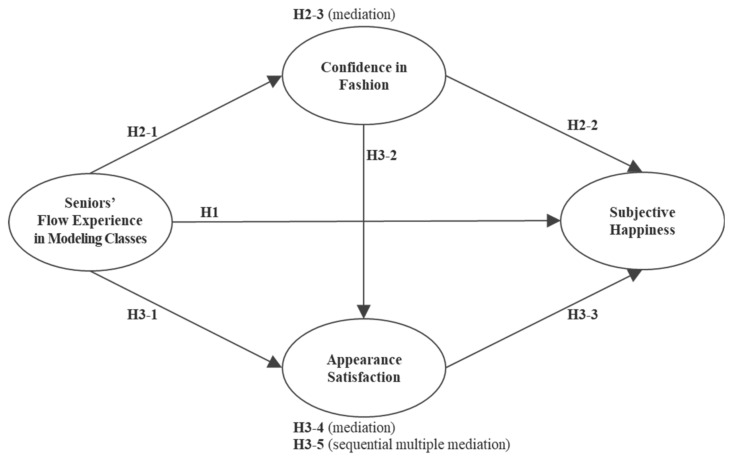
Conceptual model and hypotheses.

This study examined the influence of the seniors’ flow experience in fashion modeling classes on confidence in fashion, appearance satisfaction, and subjective happiness. The full mediation effect of confidence in fashion and appearance satisfaction were also investigated. The conceptual model is presented visually in Figure 1.

### 3.2. Measurement Scales and Data Collection

The measurement scale measures used for data collection consisted of four main variables (flow experience in fashion modeling classes, confidence in fashion, appearance satisfaction, subjective happiness) and demographics. The items measuring flow experience were revised based on Kim’s [45] questionnaire items produced by referring to the nine-dimensional characteristics of flow as claimed by Csikszentmihalyi [11]. confidence in fashion was extracted from four items related to directing confidence used in the study by An et al. [5]. Appearance satisfaction was measured through a four-item questionnaire related to attitudes and emotions toward the body and appearance with reference to Kim’s [46] research, and subjective happiness was measured through four items related to life satisfaction and evaluation used in Yoo’s study [47]. These were extracted, and the final questionnaire consisted of 16 questions. All items were modified and supplemented for use in this study and were measured on a 6-point Likert scale (1 point: not at all, 6 points: very much).

The respondents of the study were female seniors in their 50s or older who were participating in the senior fashion model training classes. An elderly modeling agency was first contacted, and participants of training classes at various levels were recruited. Empirical data collection was conducted from 17 August 2020 to 21 August 2020. Among 200 questionnaires distributed, 180 were collected and 168 were used for analysis.

### 3.3. Analysis and Respondent Characteristics

Descriptive statistics and reliabilities were conducted using SPSS version 25.0 [48]. For structural modeling, PLS-SEM (partial least squares structural equation modeling) using SmartPLS version 3.3.2 [49]. was used for this study. For the mediation effect, since indirect effects and statistical significance calculations for individual pathways can be provided and evaluated as a useful method for verifying multi-mediated effects [50], PROCESS macro version 3.5 for SPSS based on bootstrapping techniques was used [51]. The indirect effect was judged to be significant when 0 was not included in the bootstrap confidence interval.

The demographic characteristics of the respondents were as follows: 81 women (48.2%) in their 50s, 77 (45.8%) in their 60s, and 10 (6%) in their 70s or older. In terms of occupation, 31 women (18.5%) were self-employed, 30 (17.9%) were office workers or professionals, 12 (7.2%) belonged to the “other” category, and 95 women (56.6%) were unemployed or retired. The average monthly income (including unearned income) was less than KRW 1 million for 12 women (7.1%), 53 (30.9%) earned between KRW 1 million and 3 million, 48 (28.6%) earned between KRW (Korean Won) 3 million and 5 million, and 55 (32.7%) earned over KRW 5 million. Finally, 119 subjects (70.8%) had graduated from college, and 49 of them (29.2%) did not have an educational degree beyond high school.

## 4. Results and Discussion

### 4.1. Evaluation of Reliability and Validity of Reflective Measurement Models

As a result of performing the first exploratory factor analysis before analyzing the PLS-SEM, one item among the constituent items of flow experience, appearance satisfaction, and subjective happiness was excluded because it did not meet the standard for factor loading, resulting in a total of 13 items. These items were used for the final analysis. The results of evaluating the internal reliability, convergent validity, and discriminant validity of the reflective measurement model through confirmatory factor analysis are presented in Table 1 and Table 2. The outer loading of all measurement items ranged from 0.789 to 0.947 and exceeded the standard value. The results were all statistically significant at *p* < 0.001 (Table 1). In addition, the range of Cronbach’s α was between 0.797 and 0.870, which was high, and the composite reliability (CR) value was also distributed in the range of 0.881 to 0.920, proving that a high level of internal reliability was secured (Table 1). In general, the average variance extracted (AVE) reflected when evaluating the concentrated validity is 0.500 as a standard value, and when a value higher than that is obtained, it is considered to have desirable concentrated validity [52]. The AVE values of each latent variable in this study were all higher than 0.685, which indicated concentrated validity, as it exceeded the reference value (Table 1). Finally, in PLS-SEM, the Fornell–Larcker criterion [52] and approach of Henseler et al. [53] were used to determine the discriminant validity, indicating the correlation between a scale measuring a latent variable and a scale measuring another latent variable, via the heterotrait/monotrait ratio of correlations (HTMT) index. The Fornell–Larcker criterion judges that there is discriminant validity when the AVE square root value of each latent variable is larger than the correlation coefficient of other latent variables [54]. In this study, the AVE square root value for each latent variable was larger than the correlation coefficient with other latent variables, confirming discriminant validity (Table 2). Furthermore, the HTMT index was distributed in the range of 0.327 to 0.479. When the HTMT index is less than 0.850, it determines that there is discriminant validity [53]; therefore, this study meets the standard value (Table 2).

### 4.2. Evaluation of Structural Models

PLS-SEM does not yet have an international standard for the fit of a structural model and generally evaluates the fit of the model through *R*^2^ and *Q*^2^ values [54]. According to the criteria presented in previous studies, the effect of the *R*^2^ value can be classified as high for 26% or more, medium for 13% or more and 26% or less, and low for 2% or more and 13% or less [55]. In the field of consumer behavior, when the *R*^2^ value is 20% or more, predictive suitability is high [56]. The *R*^2^ values of the endogenous variables in this study (*R*^2^_confidence in fashion_ = 0.117, *R*^2^_appearance satisfaction_ = 0.222, *R*^2^_subjective happiness_ = 0.237) showed relatively low self-confidence in fashion, but appearance satisfaction and subjective happiness were higher than the standard. This structural model, according to the *R*^2^ value, was found to be suitable. The *Q*^2^ value was confirmed through the blindfolding procedure, and when the value was positive, the model was evaluated as having predictive relevance [54]. The structural model of this study was judged to have predictive suitability as all *Q*^2^ values (*Q*^2^_confidence in fashion_ = 0.069, *Q*^2^_appearance satisfaction_ = 0.168, *Q*^2^_subjective happiness_ = 0.175) exceeded the reference value of 0. In addition, the standardized root mean square residual index (SRMR), which is presented as a model fit evaluation method of PLS-SEM, was also 0.080. This satisfies the standard value of 0.080 or less, indicating that the overall model fit was secured [57,58]. Finally, looking at the internal variance inflation factor (VIF) index to determine the multicollinearity between latent variables, all values were less than the standard value of 5.00 (inner VIF: 1.000–1.285). Therefore, multicollinearity was not confirmed [56].

### 4.3. Research Hypothesis Testing

#### 4.3.1. Direct Path Verification

**Figure 2:**
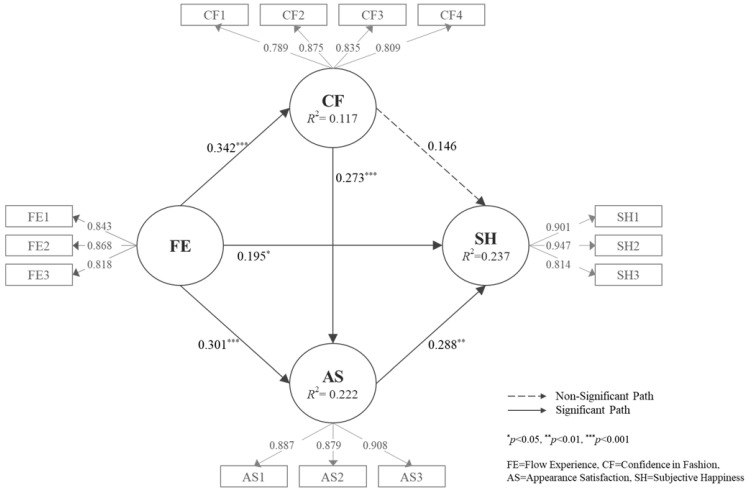
Results of structural equation modeling analysis.

A bootstrapping technique was implemented to verify the significance of the direct path. Because a minimum of 5000 resamplings is recommended for a PLS-SEM analysis, resampling was performed 5000 times [56]. The verification results are presented in Table 3 and Figure 2.

Flow experience in modeling classes was found to have a positive effect on subjective happiness (*β* = 0.195, *p* < 0.05). These results support the results of several previous studies, including those of Patall et al. [15], who reported that immersion in certain activities leads to positive emotions such as fun and affects subjective well-being [16,17,20]. Furthermore, seniors’ flow experience in modeling classes had a significant effect on confidence in fashion (*β* = 0.342, *p* < 0.001) and appearance satisfaction (*β* = 0.301, *p* < 0.01). This result is consistent with the results of a study conducted by Kim [59], who stated that fashion model flow affects fashion show confidence, and Park and Kim [9], who stated that the higher the flow experience, the higher the satisfaction. As a result, Hypotheses 1, 2-1, and 3-1 were all accepted.

The relationship between confidence in fashion and subjective happiness (*β* = 0.146, *p* = 0.105) is not statistically significant, so the results are different from those of Wood and Bandura [29] and Choi et al. [24], who reported that confidence variables are closely related to happiness. Conversely, confidence in fashion was found to have a positive effect on appearance satisfaction (*β* = 0.273, *p* < 0.001), which is in line with the results of previous studies in which stage confidence had a significant effect on satisfaction [9]. As a result, Hypothesis 2-2 was rejected, and Hypothesis 3-2 was accepted.

Finally, it was confirmed that the relationship between appearance satisfaction and subjective happiness (*β* = 0.288, *p* < 0.01) was positively significant. This supports the results of previous studies [37,41,42], which have investigated the relationship between appearance satisfaction and happiness, and Jeon [35], who examined the effect of appearance satisfaction on perceived quality of life through physical activities. Therefore, Hypothesis 3-3 is accepted.

#### 4.3.2. Simple Mediation and Multiple Mediation Effect Verification

In order to verify the simple mediation effect and multiple mediation effect of confidence in fashion and appearance satisfaction in relation to flow experience in modeling classes and subjective happiness, an analysis was conducted based on Model 6 of the SPSS PROCESS Macro. The number of bootstrap resamplings was set to 5000 in compliance with the recommended number, and the results of the mediating effect verification are presented in Table 4.

As a result of analyzing the simple mediation effect, it was found that the mediating role of confidence in fashion in the relationship between flow experience in modeling classes and subjective happiness was not significant, as can be seen from the previous direct path analysis results. In contrast, the mediating effect of appearance satisfaction was found to be significant because the 95% bootstrap confidence interval was 0.023–0.192, which did not include 0. The results were similar to those reported by Park and Chung [43]. 

In verifying the multi-mediating effect of flow experience in modeling classes on subjective happiness by sequentially mediating confidence in fashion and appearance satisfaction, the bootstrap confidence interval was 0.081–0.293, which did not include 0. Therefore, the multi-mediated effect was statistically significant. These results indicate that the influence of self-confidence in fashion, which was previously insignificant, can indirectly affect subjective happiness through appearance satisfaction.

Therefore, the indirect mediation effect of appearance satisfaction is significantly present and indirect mediation effect of confidence in fashion presents when the mediating role of appearance satisfaction exists. Hypothesis 2-3 was rejected, and Hypothesis 3-4 and 3-5 were accepted.

## 5. Conclusions 

This study focused on senior citizens who participated in fashion modeling classes for their leisure activities. Fashion model training classes recently gained popularity and are considered to have psychologically therapeutic effects on seniors. Some of the prior studies highlighted that seniors who experience modeling education showed higher self-esteem, confidence, and satisfaction than those who did not [5]. 

In this study, the flow experience, derived from the leisure activity in question, fashion model training classes, was set to be an antecedent to the perception of happiness in life. Although prior research findings on the relationship between the two variables were inconsistent, the motivation to participate in senior modeling classes was likely to be salient in the process from flow experience to subjective happiness in life. 

The empirical result of the current study confirmed the direct influence. Seniors who actively searched for modeling agencies and invested their money and effort had purposefully participated in the activities and therefore perceive the quality of their life differently. 

Fashion modeling classes involve activities related to physical attributes that elderly people are not confident in or satisfied with. Flow experience in modeling classes positively influenced confidence in fashion and appearance satisfaction. These two variables significantly mediated perceived happiness in life. One of the main findings in the mediation effect was the insights derived from the multiple serial mediation effects observed. confidence in fashion and appearance satisfaction sequentially mediated the effects of leisure flow experience on subjective happiness. This means that appearance satisfaction acts as a parameter that connects confidence in fashion and subjective happiness, confirming that there is a causal relationship in which flow experience in leisure activities sequentially mediates confidence in fashion and appearance satisfaction. The results indicate the different aspects of modeling classes that should be emphasized when managing a senior model training program. 

## 6. Suggestions

The research design of the study did not incorporate the presence of therapeutic effects of fashion modeling classes for elderly people. However, study results could be applied to therapeutic practices because if senior fashion model trainees could experience a higher level of flow in leisure activities, they might be more confident in their fashion senses (critical to their activity performance) and more satisfied with their appearances (critical to eliminating depression caused by physical changes with aging). Ultimately, the individual’s feeling of happiness in life would be increased. In addition, as social interest in the effects of leisure activities for seniors has increased in recent years, practitioners and social works could develop diverse educational leisure programs by implementing the findings of the current study. 

This study provided several insights into academic research in that it identified that flow experience, which has been mainly used as a mediating variable, could exert an independent influence. In addition, the causation from flow experience, self-confidence, satisfaction, and subjective happiness was confirmed through a multi-mediated effect analysis. Most fashion therapy research focused on the effect of changing a patient’s appearance on patients’ mental health. This study expanded the scope of the research agenda on perceived happiness in life, which is critical to the mental health of an increasing number of elderly populations.

To eliminate external influences such as gender effects, this study was conducted on a female sample. Futures studies should incorporate gender differences in fashion modeling classes, as male and female seniors differ in their experiences with fashion products and level of flow experience in training classes. For a better understanding of the therapeutic effects of fashion modeling programs, a research design with a control group should be further considered. Other variables, such as the number of educational programs a person is enrolled in or people’s use of in fashion products, should be incorporated in the future studies as well. Social variables such as the interaction with program instructors require further investigation for more theoretical findings.

## Figures and Tables

**Table 1:** Results of reliability and confirmatory factor analysis.

Constructs	Items	Outer Loading	Cronbach’s α	CR	AVE
Seniors’ Flow Experience In Modeling Classes	I am unaware of the passage of time during model class.	0.843 ***	0.797	0.881	0.711
	I come to forget all my worries during model class.	0.868 ***			
	I am completely immersed in the fashion model education I am taking.	0.818 ***			
Confidence in Fashion	I think that, despite wearing the same clothes, I look nicer than other people when I wear them.	0.789 ***	0.853	0.897	0.685
	I can harmoniously coordinate various clothes.	0.875 ***			
	I know how to choose and wear clothes that suit me well.	0.835 ***			
	When buying clothes, I can choose colors that go well with each other.	0.809 ***			
Appearance Satisfaction	I am satisfied with my body proportions.	0.887 ***	0.870	0.920	0.794
	I like how I look in the mirror.	0.879 ***			
	I am satisfied with my appearance.	0.908 ***			
Subjective Happiness	I am satisfied with my life now.	0.901 ***	0.866	0.918	0.790
	I am enjoying my life now.	0.947 ***			
	I am living the life I have dreamed of so far.	0.814 ***			

*** *p* < 0.001.

**Table 2:** Assessment of discriminant validity.

Constructs	Fornell–Larcker Criterion				Heterotrait/Monotrait Ratio (HTMT)			
	FE	CF	AS	SH	FE	CF	AS	SH
Flow Experience	**0.843** ^a^				-			
Confidence in Fashion	0.342 ^b^	**0.828**			0.388	-		
Appearance Satisfaction	0.395	0.376	**0.891**		0.474	0.397	-	
Subjective Happiness	0.358	0.321	0.420	**0.889**	0.423	0.372	0.479	-

FE = flow experience, CF = confidence in fashion, AS = appearance satisfaction, SH = subjective happiness; ^a^: bold numbers represent the square root of AVE for constructs; ^b^: numbers below the diagonal are correlation estimates between two constructs.

**Table 3:** Results of direct path coefficient verification.

Hypothesis	Direct Paths	*β*	S.E.	*t*	Results
H1	FE → SH	0.195	0.091	2.148 *	Accepted
H2-1	FE → CF	0.342	0.078	4.405 ***	Accepted
H2-2	CF → SH	0.146	0.090	1.623	Rejected
H3-1	FE → AS	0.301	0.092	3.280 ***	Accepted
H3-2	CF → AS	0.273	0.073	3.747 ***	Accepted
H3-3	AS → SH	0.288	0.095	3.024 **	Accepted

* p < 0.05, ** *p* < 0.01, *** *p* < 0.001; FE = flow experience, CF = confidence in fashion, AS = appearance satisfaction, SH = subjective happiness.

**Table 4:** Results of multiple mediation effect analysis.

Hypothesis	Indirect Paths	*β*	S.E.	95% Confidence Interval		Results
				LLCI	ULCI	
H2-3	FE → CF → SH	0.055	0.039	−0.006	0.143	Rejected
H3-4	FE → AS → SH	0.095	0.043	0.023	0.192	Accepted
H3-5	FE → CF → AS → SH	0.026	0.016	0.081	0.293	Accepted

FE = flow experience, CF = confidence in fashion, AS = appearance satisfaction, SH = subjective happiness.

## Data Availability

Data are not publicly available, although they may be made available on request from the corresponding author.
